# Perlecan in the Natural and Cell Therapy Repair of Human Adult Articular Cartilage: Can Modifications in This Proteoglycan Be a Novel Therapeutic Approach?

**DOI:** 10.3390/biom11010092

**Published:** 2021-01-13

**Authors:** John Garcia, Helen S. McCarthy, Jan Herman Kuiper, James Melrose, Sally Roberts

**Affiliations:** 1 School of Pharmacy and Bioengineering, Keele University, Newcastle-under-Lyme, Staffordshire ST5 5BG, UK; john.garcia@nhs.net (J.G.); helen.mccarthy6@nhs.net (H.S.M.); jan.kuiper@nhs.net (J.H.K.); 2 Spinal Studies & Cartilage Research Group, Robert Jones and Agnes Hunt Orthopaedic Hospital NHS Foundation Trust, Oswestry, Shropshire SY10 7AG, UK; 3 Raymond Purves Bone and Joint Research Laboratory, Kolling Institute of Medical Research, Northern Sydney Area Local Health District, St. Leonards, NSW 2065, Australia; james.melrose@sydney.edu.au; 4 Sydney Medical School, Northern, The University of Sydney, Faculty of Medicine and Health, Royal North Shore Hospital, St. Leonards, NSW 2065, Australia; 5 Graduate School of Biomedical Engineering, Faculty of Engineering, University of New South Wales, Sydney, NSW 2052, Australia

**Keywords:** human articular cartilage, perlecan, heparan sulphate, heparanase, cartilage repair, natural repair, chondrocytes

## Abstract

Articular cartilage is considered to have limited regenerative capacity, which has led to the search for therapies to limit or halt the progression of its destruction. Perlecan, a multifunctional heparan sulphate (HS) proteoglycan, promotes embryonic cartilage development and stabilises the mature tissue. We investigated the immunolocalisation of perlecan and collagen between donor-matched biopsies of human articular cartilage defects (*n* = 10 × 2) that were repaired either naturally or using autologous cell therapy, and with age-matched normal cartilage. We explored how the removal of HS from perlecan affects human chondrocytes in vitro. Immunohistochemistry showed both a pericellular and diffuse matrix staining pattern for perlecan in both natural and cell therapy repaired cartilage, which related to whether the morphology of the newly formed tissue was hyaline cartilage or fibrocartilage. Immunostaining for perlecan was significantly greater in both these repair tissues compared to normal age-matched controls. The immunolocalisation of collagens type III and VI was also dependent on tissue morphology. Heparanase treatment of chondrocytes in vitro resulted in significantly increased proliferation, while the expression of key chondrogenic surface and genetic markers was unaffected. Perlecan was more prominent in chondrocyte clusters than in individual cells after heparanase treatment. Heparanase treatment could be a means of increasing chondrocyte responsiveness to cartilage injury and perhaps to improve repair of defects.

## 1. Introduction

Articular cartilage can withstand compressive, tensile and shear loading and provides efficient articulation of diarthrodial joints. If left untreated, damaged articular cartilage in a joint can lead to osteoarthritis (OA) and ultimately joint failure [1,2]. Cell-based therapies have been developed to promote cartilage repair and the regeneration of complex articular structure to help patients with damaged or degenerate cartilage [3,4].

It is commonly reported that adult articular cartilage has a limited capacity for self-regeneration [5]; however, a growing body of evidence from in vitro and in vivo models suggests that in some cases, cartilage can undergo some form of natural repair [6,7]. A bovine explant model of cartilage healing showed that both young and mature animals produced an outgrowth of tissue from the artificially damaged sites, but with young tissues generating more hyaline-like cartilage [6]. In humans, magnetic resonance imaging (MRI) observation of the knees of healthy subjects showed that some cartilage defects (tibial and patellar) reduced in size or were completely filled between a baseline scan and a two year follow up [7]. A natural healing response was also seen in some cartilage lesions of subjects with anterior cruciate ligament (ACL) injuries 6–56 months after reconstructive surgery to repair the ligament damage [8]. The mechanisms by which articular cartilage repairs itself is poorly understood, but is believed to involve an interplay between cellular, biochemical and mechanical factors [9,10,11].

Perlecan, also known as heparan sulphate proteoglycan-2, is a modular, multifunctional proteoglycan with an ability to promote chondrocyte proliferation, differentiation and matrix synthesis through its interactions with a large repertoire of ligands including growth factors, morphogens and extracellular matrix (ECM)-stabilising glycoproteins [12,13]. One of the glycosaminoglycans contained in perlecan, heparan sulphate (HS), is a vital extracellular component. Its cleavage causes matrix remodelling through the release of HS-bound cytokines, growth factors, morphogens, proteases and inhibitory proteins which regulate many cellular pathological and physiological processes [14,15]. Perlecan, through its HS chains, has chondrogenic properties and is able to regulate cell signalling, matrix assembly and new tissue formation [12,16,17]. These attributes make perlecan an important candidate molecule when trying to understand how cartilage repairs itself. Hence, harnessing these attributes could also be beneficial in promoting the repair of damaged articular cartilage in human joints. Heparanase is an endo-β-glucuronidase cleaving the β(1,4)-glycosidic linkages between GlcN and GlcA in heparan sulphate (HS), and is the only known mammalian enzyme displaying this glycolytic activity [18].

Interactions between perlecan and collagen type VI have been well established [19] and, like perlecan, collagen type VI is believed to be involved in chondrocyte adhesion, integrity and matrix interactions [20,21]. Collagen type III is another minor collagen found in articular cartilage and has been suggested to have a role in reinforcing the cartilage matrix as part of a healing response to matrix damage [22,23].

In the present study, we have immunolocalised perlecan and types III and VI collagen for the first time in donor-matched samples of naturally and cell therapy repaired articular cartilage of the human knee. We have also investigated whether the phenotype and proliferation of cultured human chondrocytes was affected by the removal of cell surface HS. We hypothesise that the distribution of perlecan in repaired adult cartilage mimics its distribution in embryological cartilage.

## 2. Materials and Methods

### 2.1. Tissue Samples and Histology

The National Research Ethics Service (11/NW/0875) gave ethical approval and informed written consent was obtained from patients undergoing autologous cell therapy for cartilage defects in their knee (*n* = 10, aged 29–51 years). This procedure entails harvesting ~200 mg macroscopically healthy cartilage, usually from the trochlea, from which chondrocytes are isolated and culture expanded in monolayer, prior to re-implantation in the defect site, usually on the patella or lateral/medial femoral condyles (LFC/MFC) [3]. At approximately 12 months post-implantation, full-depth cartilage biopsies with subchondral bone (1.8 mm diameter) were obtained using a juvenile bone-marrow biopsy needle from both the harvest site (naturally repaired) and the defect site where the cells had been implanted (cell-treated repair). The location of these was ensured via the use of knee maps [24], where the location of each procedure is recorded at the time of original surgery. Macroscopically healthy cartilage was also obtained from the knees of five cadavers (aged 21–63 years) and four donors undergoing total knee arthroplasty for OA (aged 51–81 years). A description of the donor demographics and tissue samples used in the following experiments can be found in Table 1. Healthy cadaveric cartilage from donors 11–14 was obtained within 24 h of death from the UK Human Tissue Bank with approval by the Trent Research Ethics Committee (UK). Full-depth core biopsies of other samples (from TKR and natural and cell therapy repair patients) of cartilage and underlying bone were snap frozen within 2–4 h of harvesting in liquid nitrogen-cooled hexane and stored at −196 °C until cryosectioning. Cores were embedded into tissue-freezing medium (Leica) and cryosectioned at 7 µm thickness onto poly-L-lysine-coated slides. Cryosections were then stained with either haematoxylin and eosin (H&E) or toluidine blue for the assessment of general morphology and proteoglycan content of the cartilage, respectively. Collagen fibre organisation and orientation were assessed under polarised light. The quality of the repaired cartilage was assessed and scored semi-quantitatively using both the International Cartilage Repair Society II Histology Score (ICRS II) [25] and the Oswestry Score [26], where a higher score in each system represents better-quality cartilage.

### 2.2. Immunohistochemistry

Cryosections were brought to room temperature and treated with 4800 U/mL hyaluronidase (Sigma, Merck Life Science UK, Dorset, UK) for 2 h and fixed with 4% formaldehyde for 10 min. Slides were washed 3 times in phosphate buffered saline (PBS) between all steps and all steps were performed at room temperature. Goat and horse serum were used to block non-specific binding of the primary mouse and rabbit antibodies, respectively (30 min). Sections were then incubated with mouse monoclonal primary antibodies against perlecan (clone A74, Abcam, Cambridge, UK), collagen type III (clone FH-7A, Abcam) and a polyclonal rabbit antibody to bovine collagen type VI (kindly gifted by Shirley Ayad, University of Manchester, UK) for 60 min, then incubated with biotinylated goat anti-mouse and horse anti-rabbit secondary antibodies (Vectastain Elite ABC kit, Vector Laboratories, Upper Heyford, UK) for monoclonal and polyclonal primary antibodies, respectively, for 30 min. An isotype-matched IgG was used in place of the primary monoclonal antibodies (R&D, Cat No MAB002) as a negative control and normal rabbit serum (Abcam, Cat no ab7487) for the polyclonal, and 0.3% hydrogen peroxide in methanol was used to block endogenous peroxidase activity (30 min). The Vectastain Elite ABC kit (Vector Laboratories) was used to enhance labelling and the ImmPACT^®^ DAB Peroxidase substrate (Vector Laboratories) was used to reveal staining. The sections were dehydrated in serial solutions of 70%, 90% and 100% isopropanol (2 min each) and cleared in xylene (2 × 5 min). The slides were mounted in Pertex (CellPath, Newtown, UK) before imaging.

A semi-quantitative score was developed to assess the immunolocalisation and degree of staining for perlecan in the superficial, mid, and deep zones of the cartilage biopsies. Each zone was scored separately as 0 = no staining, 1 = pericellular staining, 2 = mixture of pericellular and matrix staining, or 3 = matrix staining. Each sample was then given an overall score which was a summation of the scores for the three zones. A high overall score equates to a more widespread matrix immunostaining, whereas a low score equates to more restricted pericellular staining throughout the tissue. Image analysis was performed using FIJI-ImageJ software (Version 1.5), using the Colour Deconvolution and Threshold plugins to establish the levels of perlecan staining as a percentage of the total area of the section.

### 2.3. Isolation and Culture of Chondrocytes

Chondrocytes were isolated from macroscopically normal cartilage taken from four patients having arthroplasty and one cadaver (Table 1), as previously described [27]. In brief, cartilage tissues were minced and digested for 16 h with collagenase type II (250 IU/mg dry weight, Worthington, New Jersey, USA) at 37 °C. The extracted cells, were seeded at 5000 cell/cm^2^ in complete culture media containing Dulbecco’s Modified Eagle’s Medium/F-12 (DMEM/F-12) with 1% (*v*/*v*) penicillin/streptomycin (P/S) and 10% (*v*/*v*) foetal calf serum (all Life Technologies, Loughborough, UK). Chondrocytes were passaged at 70–80% confluence and cultured to passage 2 (P2).

### 2.4. Heparanase Treatment of Chondrocytes and Live Cell Imaging

At P2, chondrocytes were seeded into 12-well plates at or in chamber slides (with 8 chambers) 5200 cells/cm^2^, and treated with complete media supplemented with or without 200 ng/mL of recombinant active human heparanase (Bio-Techne, Abingdon, UK; 20 ng of enzyme results in >50% of optical density (OD) reduction as measured by heparan sulphate release from human syndecan-4) for 48 h. The 12-well plate was placed in a Cell-IQ (ChipMan Technologies, Tampere, Finland) live imaging platform to acquire phase contrast images of all wells, every ten minutes, during the 48-h culture. A built-in analysis software in the Cell-IQ was used to determine the number of cells in each image to produce growth curves of cells treated with heparanase, in comparison to control cells with no enzyme. The mean and standard deviation of the cell counts from three fields of view from three separate repeat wells were taken. After 48 h, the cells were harvested and prepared for multichromatic flow cytometry and real-time quantitative polymerase chain reaction (RT-qPCR) analysis.

The cells within the chamber slides were washed three times with PBS, fixed with paraformaldehyde for 10 min and chamber slides were stored at 4 °C until used for immunocytochemistry.

### 2.5. Immunocytochemistry and Toluidine Blue Staining of Heparanase-Treated Chondrocytes

Chamber slides were brought to room temperate and the PBS replaced with 0.2% Tween 20 for 10 min to permeabilise the cells. After three washes with PBS, the same staining protocol used for immunohistochemistry (see Section 2.2) was followed to reveal the presence of perlecan on the adherent cells, with the addition of a haematoxylin counterstain (diluted 1:3) for 5 s before the slides were mounted in Pertex.

To visualise the presence of glycosaminoglycans, chamber slides were brought to room temperate and the PBS replaced with toluidine blue for 30 s, then washed with distilled water for 5 min. The slides were dehydrated in 70%, 90% and 100% isopropanol (2 min each) and cleared in xylene (2 × 5 min). The slides were mounted in Pertex for imaging.

### 2.6. Multichromatic Flow Cytometry

A panel of 12 surface markers was used in multichromatic flow cytometry to assess the phenotype of the cells. The harvested cells were blocked with human IgG (Grifols, Cambridge, UK) for 1 h, washed with PBS and incubated for 30 min with antibodies against the mesenchymal stromal/stem cell (MSC) markers CD73, CD90 and CD105 putative chondropotency markers CD151, CD166, FGFR3, CD44 and integrins CD29, CD49a, CD49b CD49c, CD51/CD61 (all BD Biosciences, except for FGFR-3 which was sourced from R&D Systems). The matching isotype controls for each antibody were also prepared according to manufacturer’s recommendations. At least 5000 cells were measured per marker via a FACS Canto II cytometer and analysis was performed using the FACS Diva software.

### 2.7. RNA Extraction and Reverse Transcription Quantitative Polymerase Chain Reaction (RT-qPCR)

To determine the effects of the heparanase treatment on gene expression, RNA was extracted using the RNeasy^®^ mini kit (Qiagen, Manchester, UK) and cDNA was generated using a High-Capacity cDNA Reverse Transcriptase Kit^®^ (Applied Biosystems, Loughborough, UK) according to the manufacturers’ protocols. RT-qPCR was performed on a QuantStudio 3 real-time PCR system (Applied Biosystems) using SYBR green QuantiTect primer assays (Qiagen) to assess the gene expression of Sox-9 (*SOX9*), aggrecan (*ACAN*), collagen type II (*COL2A1*), fibroblast growth factor receptor 3 (*FGFR3*), collagen type X (*COL10*) and activin receptor-like kinase (*ALK-1*). Peptidylprolyl Isomerase A (*PPIA*) and TATA-box binding protein (*TBP*) were used as reference genes and the delta-delta C_t_ method was employed to determine the relative fold change in gene expression levels between heparanase-treated and untreated cells.

### 2.8. Statistical Analysis

Statistical analysis was performed using GraphPad Prism version 7. The Shapiro–Wilk test was used to determine the normality of data. *T*-tests and Pearson’s test were used to compare and correlate histology and immunohistochemistry scores, respectively. A two-way ANOVA with multiple comparisons was used to analyse the growth kinetics of the cells treated with heparanase and a paired, one-sample *t*-test for the fold change in gene expression. A *p*-value ≤ 0.05 was considered statistically significant.

## 3. Results

### 3.1. Morphological Structure of Healthy and Repaired Cartilage

**Figure 1:**
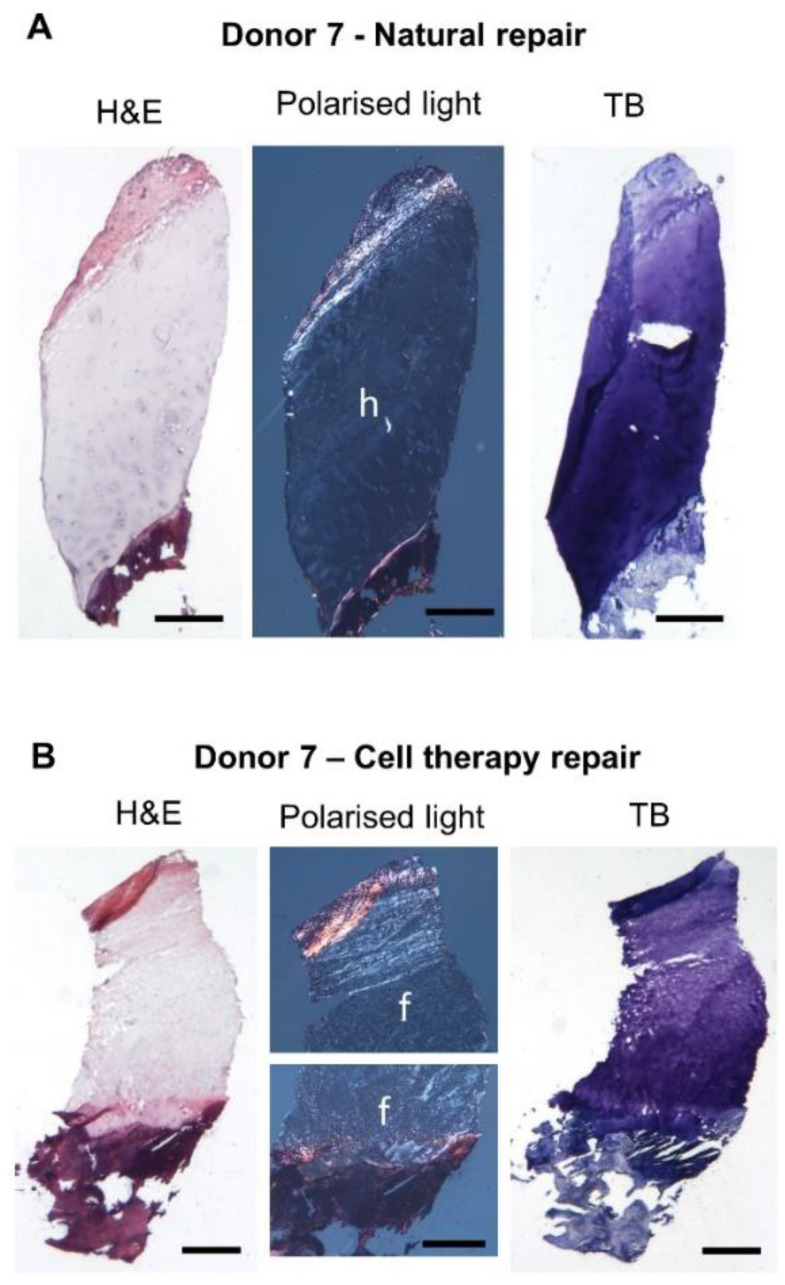
Representative histology images of cartilage repair biopsies from the same donor. Natural repair (**A**) and cell therapy repair (**B**) cryosections were stained with haematoxylin and eosin (H&E) to assess general morphology and toluidine blue (TB) to assess proteoglycan content; both samples demonstrated good to excellent matrix metachromasia. Polarised light was used to assess collagen fibre orientation and determine tissue morphology. The natural repair cartilage demonstrated a mostly hyaline (h) morphology whilst the cell therapy repair cartilage was mostly fibrocartilage (f). Scale bars 500 μm.

The general morphology of the repaired tissue biopsies was very variable, more so for the naturally repaired samples than the cell therapy repaired samples. Overall, donor-matched natural and cell therapy repaired samples showed no distinguishable trend or correlation in terms of tissue morphology (Figure 1). Of the naturally repaired biopsies, 3/10 were predominantly hyaline and 4/10 fibrocartilage, 1/10 was a mixture of hyaline and fibrocartilage and 2/10 were a fibrous morphology. Of the cell therapy repaired biopsies, 7/10 were fibrocartilage and 3/10 were of a mixed hyaline/fibrocartilage morphology with no discernible differences in tissue morphology noted with varying anatomical location of the repair cartilage site. The ICRS overall histology score was not significantly different between naturally repaired and cell therapy repaired samples (mean scores of 5.6 ± 1.9 SD and 5.1 ± 0.8 SD, respectively, *p* = 0.393). Matrix metachromasia was generally better in the cell therapy repaired cartilage samples than in the naturally repaired ones. Cell morphology was marginally better in the cell therapy repaired biopsies, but not significantly different to the naturally repaired biopsies. Vascularisation was observed in 6/10 naturally repaired biopsies, but not in the cell therapy repaired or normal samples.

### 3.2. Perlecan and Collagen Types III and VI Have a Diffuse Immunolocalisation in Repair Cartilage Tissues

**Figure 2:**
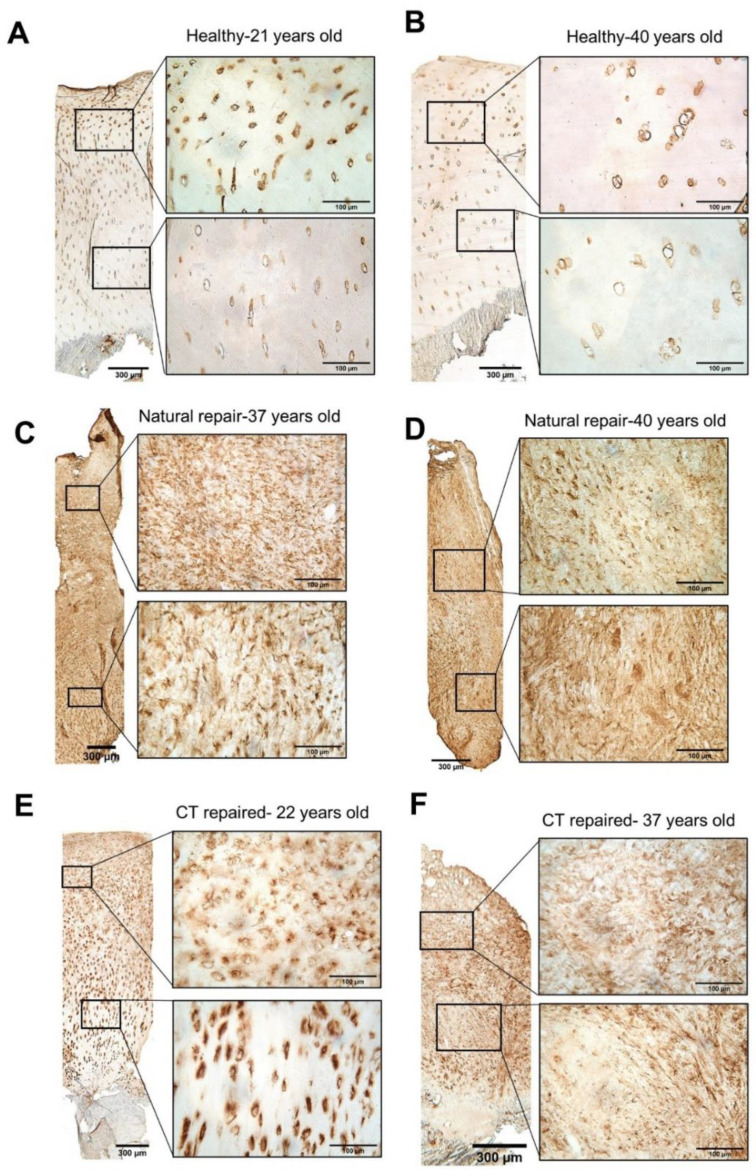
Immunohistochemistry of perlecan. Monoclonal antibodies (A74) were used to detect the presence of perlecan in cryosections of core biopsies. (**A**,**B**) Heathy cartilage (*n* = 5) from cadavers all showed distinct pericellular staining for perlecan with a typically hyaline morphology. (**C**,**D**) Naturally repaired cartilage (*n* = 10) from the harvest site of autologous cell therapy donors showed heterogenous staining patterns, some having both widespread matrix and pericellular staining ((**C**), donor 10), whilst in others there was diffuse matrix staining throughout ((**D**), donor 9). (**E**,**F**) Cell therapy repaired (CT, *n* = 9) cartilage also showed a heterogenous localisation for perlecan, similar to the naturally repaired tissues. The sample depicted in (**E**) (donor 2) shows pericellular staining for perlecan in repair tissue with hyaline cartilage morphology, but not as discretely as in the healthy tissues. The sample depicted in (**F**) (donor 7) shows predominantly matrix immunolocalisation of perlecan. Scale bars show 300 µm for low magnification images and 100 µm for high magnification inserts. Isotype controls found in Supplementary Figure S1A–C.

Perlecan was localised in a discrete manner in the pericellular matrix around chondrocytes in healthy cartilage (Figure 2A,B). However, in naturally and cell therapy repaired cartilage staining for perlecan was seen in a pericellular location in some biopsies, diffusely throughout the matrix in others or both patterns within others. Where fibrocartilage was more abundant, perlecan was more diffuse in the cartilage matrix with some strong staining around chondrocytes, which was strikingly different to healthy cartilage as illustrated in Figure 2C,D, showing donors 10 and 9, respectively. In both natural and cell therapy repaired tissues where hyaline cartilage was visible, perlecan was mostly localised in the pericellular regions, but more prominently than in normal cartilage (Figure 2E, showing donor 2). The more elongated cells within fibrocartilaginous repair tissue were generally weak or moderately stained for perlecan, compared to the more rounded chondrocytes in hyaline cartilage (both repair and normal cartilage) which had strong pericellular perlecan immunostaining. Disorganised fibrous tissue was associated with weak matrix perlecan staining. Isotype controls are shown in Figure S1.

**Figure 3:**
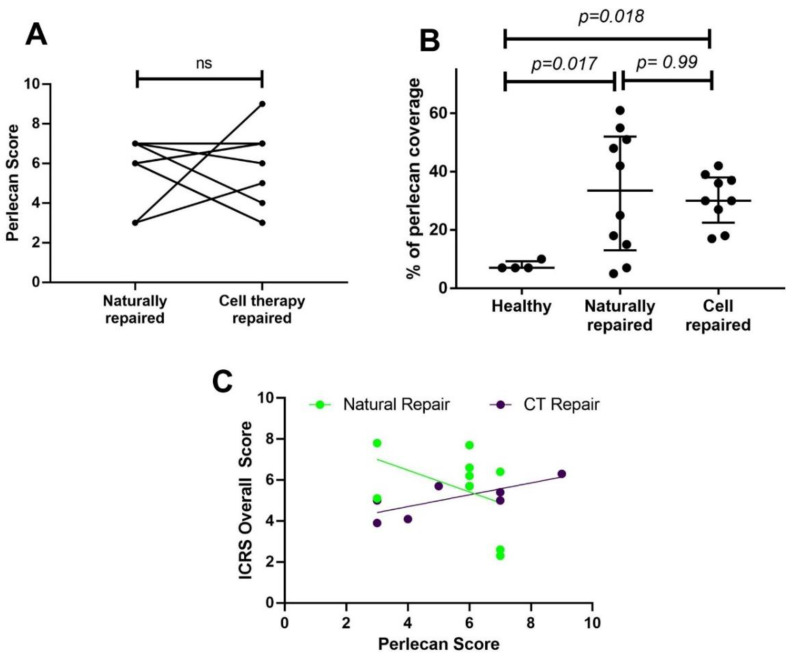
Analysis of tissue morphology and perlecan staining. (**A**) The perlecan immunohistochemistry score gives a general idea of the localisation (pericellular, non-pericellular, mixed) of perlecan in the deep, middle and superficial zones of cartilage. The zones were scored as 0 = no staining, 1 = pericellular staining, 2 = mixture of pericellular and matrix staining, or 3 = matrix staining. The final perlecan score shown here is the summation of the scores for the three zones in each sample. No difference was found between the donor-matched natural and cell therapy repaired tissues. Data show the median with interquartile range. (**B**) Threshold image analysis confirmed a higher percentage of perlecan staining in naturally repaired and CT repaired cartilage than in health cartilage. Perlecan was significantly more prominent in the repair tissues compared to controls. (**C**) Regression analysis showed a positive correlation between the ICRS score and perlecan immunohistochemical score (*r* = 0.75, *p* = 0.03) for cell therapy repaired, but not naturally repaired tissues (*r* = −0.4, *p* = 0.25).

The perlecan immunohistochemistry scores were similar between the two repair tissues, with no noticeable trend when comparing individual donor-matched samples (Figure 3A). Image analysis of the percentage of perlecan staining in the tissues showed that naturally repaired and cell therapy repaired cartilage had significantly more staining than the healthy tissues (*p* = 0.017 and *p* = 0.018, respectively, Figure 3B). Interestingly, an increase in the perlecan score significantly correlated with a better-quality cell therapy repair, as defined by the ICRS II ‘overall score’ parameter (r = 0.75, *p* = 0.03, Figure 3C). Perlecan was also strongly localised around small blood vessels that were visible in 6 of the 10 naturally repaired. No blood vessels were observed in either the cell therapy repaired cartilage samples, or the healthy cartilage.

**Figure 4:**
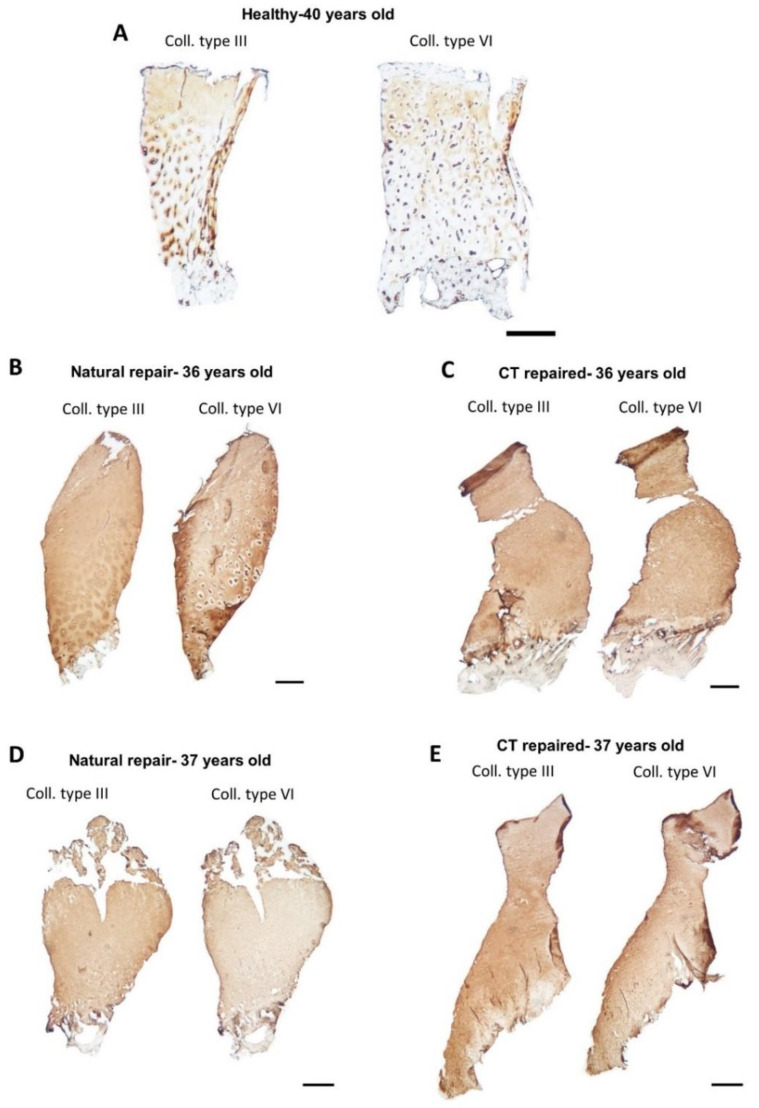
Immunohistochemistry of collagen types III and VI. Monoclonal and polyclonal antibodies were used to detect the presence of collagens type III and VI, respectively, in cryosections of core biopsies. For the repair tissues, two donor-matched samples of natural and cell therapy (CT) repaired cartilage are shown as representative examples (B + C = donor 7, D + E = donor 9). (**A**) Healthy cartilage showing interterritorial staining for collagen type III and pericellular staining for type VI (donor 15). (**B**,**C**) In this instance of hyaline-like cartilage in naturally repaired cartilage, the collagen type III was localised in the interterritorial region while collagen type VI was localised in the pericellular matrix. (**D**,**E**) Both collagens type III and VI are diffused in the matrix of fibrocartilage. Scale bar = 500 µm. Isotype controls found in Figure S1D,E.

Collagen types III and VI generally exhibited a diffuse staining pattern throughout the interterritorial matrix, covering 94.3 ± 8.9% (range 70–100) and 95.2 ± 7.1% (range 80–100) of the section area, respectively (Figure 4C,D). However, where there was hyaline cartilage present in the repair tissues (Figure 4B), the staining pattern in these regions for both collagen types III and VI was similar to what is typically observed in healthy cartilage (Figure 4A) [28,29], with the pericellular matrix being immunonegative for collagen type III and immunopositive for collagen type VI and the territorial matrix being immunopositive for collagen type III and immunonegative for collagen type VI.

### 3.3. Heparanase Increases the Proliferation of Chondrocytes

**Figure 5:**
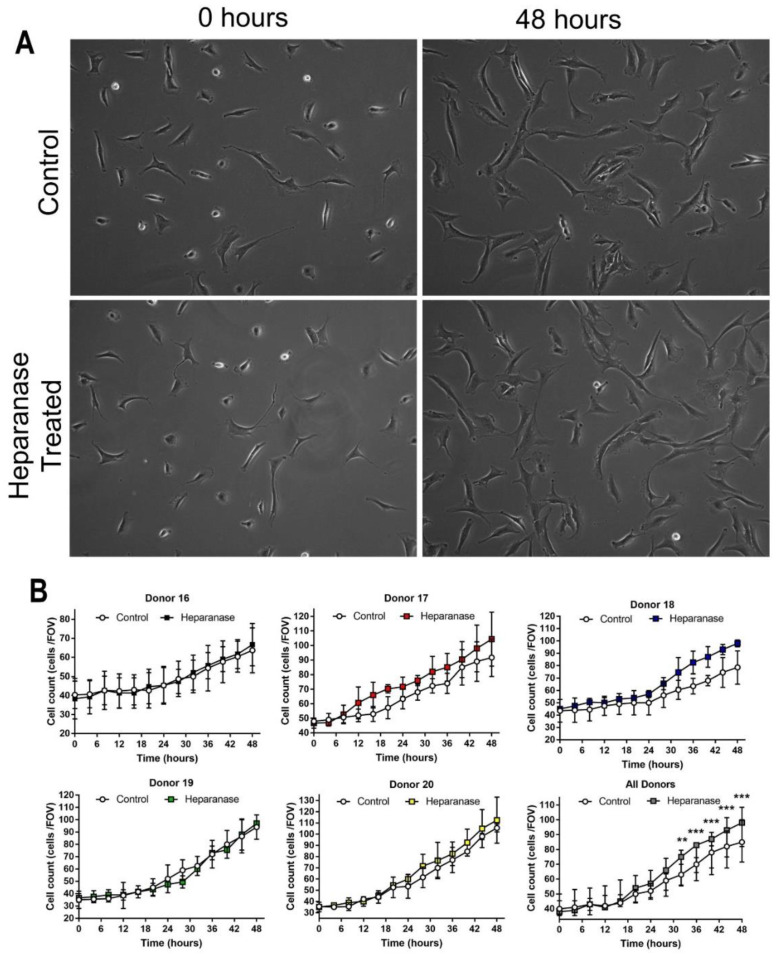
Morphology and growth kinetics of chondrocytes after treatment with heparanase (*n* = 5). (**A**) Phase contrast images were acquired for control and heparanase-treated chondrocytes every 10 min at precise locations for 48 h. The representative images shown are of chondrocytes from donor 20 at t = 0 h and t = 48 h. (**B**) Growth kinetics of chondrocytes were established using a live cell imaging platform and analysis software during the 48 h heparanase treatment period. Individual plots are shown for donors 16 to 20 with mean and SD of cell counts from three FOV from three separate wells. The combined data for all five donors at every time point are also shown (bottom right). FOV = field of view. ** *p* < 0.05, *** *p* < 0.01.

No discernible difference in morphology was observed in chondrocytes cultured in monolayer which had been treated with 200 ng/mL of heparanase compared to untreated controls after 48 h (Figure 5A). Separate and combined growth plots are shown for the individual donor cell populations tested in Figure 5B. Whilst there is variation between donors, a combined assessment of the cell populations showed that, for the first 20 h, the heparanase-treated and control chondrocytes showed similar growth rates, but diverged from 24 h onwards with treated cells showing significantly higher proliferation rates than untreated control cells between 32 and 48 h (Figure 5B, bottom right plot).

### 3.4. Stromal/Stem Cell and Chondropotency Markers and Genes Are Not Affected by Heparanase

**Figure 6:**
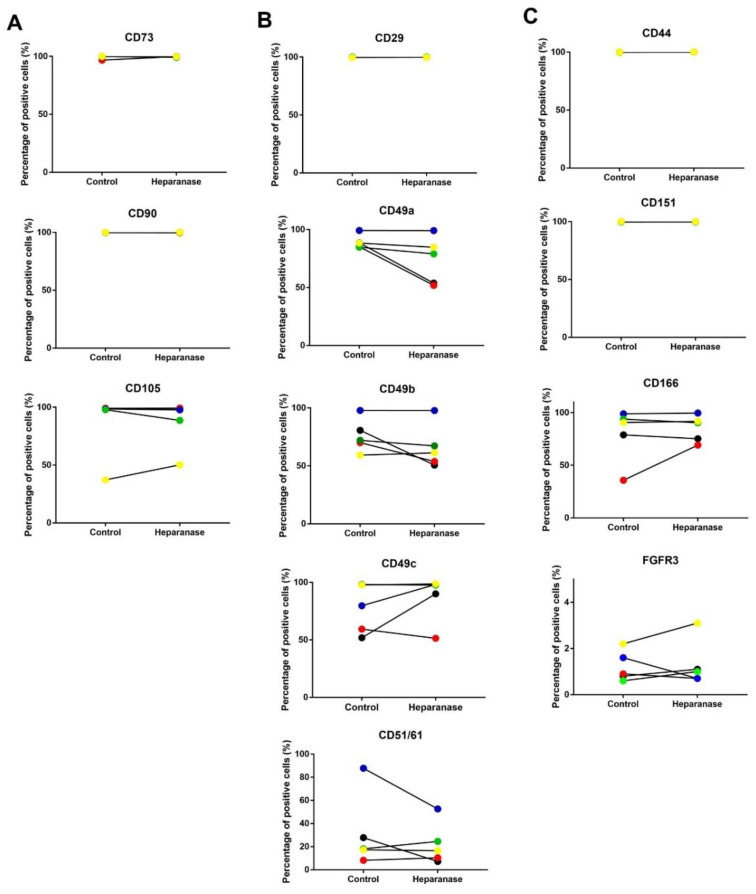
Flow cytometry analysis of the effects of heparanase treatment on surface markers of chondrocytes (*n* = 5). Results are shown as the percentage of positive cells for a particular marker on heparanase-treated chondrocytes and the matching control. Fluorochrome-conjugated antibodies were used to detect (**A**) stem cells markers, (**B**) integrins and (**C**) chondrogenic markers. No significant differences were observed. Matched samples are represented by the same colour dot; donor 16 (black), donor 17 (red), donor 18 (blue), donor 19 (green), and donor 20 (yellow).

Flow cytometry demonstrated that the positivity of stromal/stem cell markers, CD73, CD90 and CD105 (Figure 6A), and the chondrogenic markers CD44, CD151, CD166, FGFR3 were unaffected by heparanase treatment (Figure 6B), although CD166 and FGFR3 showed a high level of variability between donors. For the integrins, donor variability was also observed with CD49a, CD49b, CD49c and CD51/61, but not CD29, with no statistical difference between treated cells and controls for any of the integrins (Figure 6C). Chondrocytes from donor 17, the oldest donor, showed a marked heparanase-induced increase in CD166 and a noticeable decrease in CD49a, CD49b and CD49c compared to the cells from the other donors.

**Figure 7:**
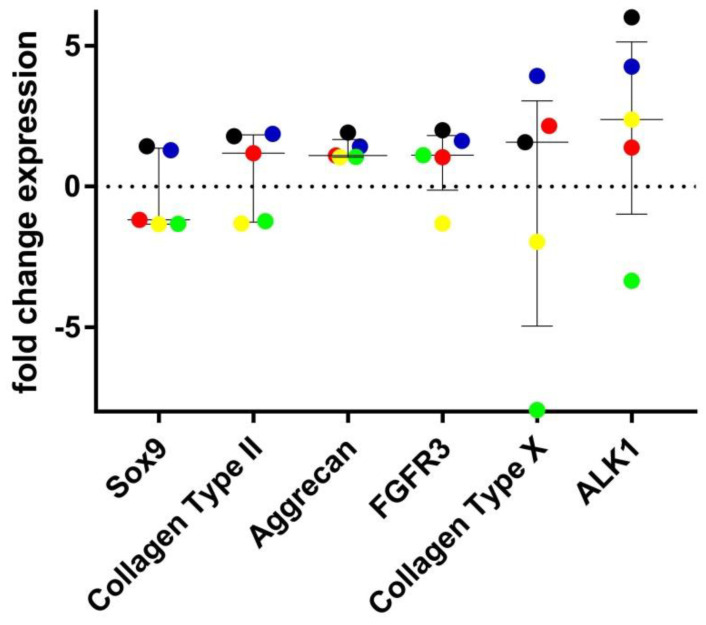
Analysis of the effects of heparanase treatment on gene expression of chondrocytes in monolayer culture (*n* = 5). Results are presented as log-fold change in the expression of the chondrogenic genes SOX9, collagen type II, aggrecan, FGFR3 and hypertrophy genes collagen type X and ALK1 in chondrocytes that were treated with heparanase compared to the untreated controls. Matched samples are represented by the same colour dot; donor 16 (black), donor 17 (red), donor 18 (blue), donor 19 (green), and donor 20 (yellow). Error bars indicate medians and interquartile ranges.

Although the relative fold change in chondrogenic gene expression was not statistically significant between the heparinase-treated and untreated chondrocytes, there was a general decrease in SOX9 expression (median= −1.17), and increased expressions for ACAN (median = 1.1), COL2A1 (median =1.2), and FGFR3 (median = 1.1) following heparanase treatment (Figure 7). The relative fold change in expression of the hypertrophic genes COL10 (median = 1.6) and ALK-1 (median= 2.4) was also increased following heparanase treatment, but this was not statistically significant (Figure 7).

### 3.5. Perlecan and Toluidine Blue Staining Is More Prominent in Chondrocyte Clusters

**Figure 8:**
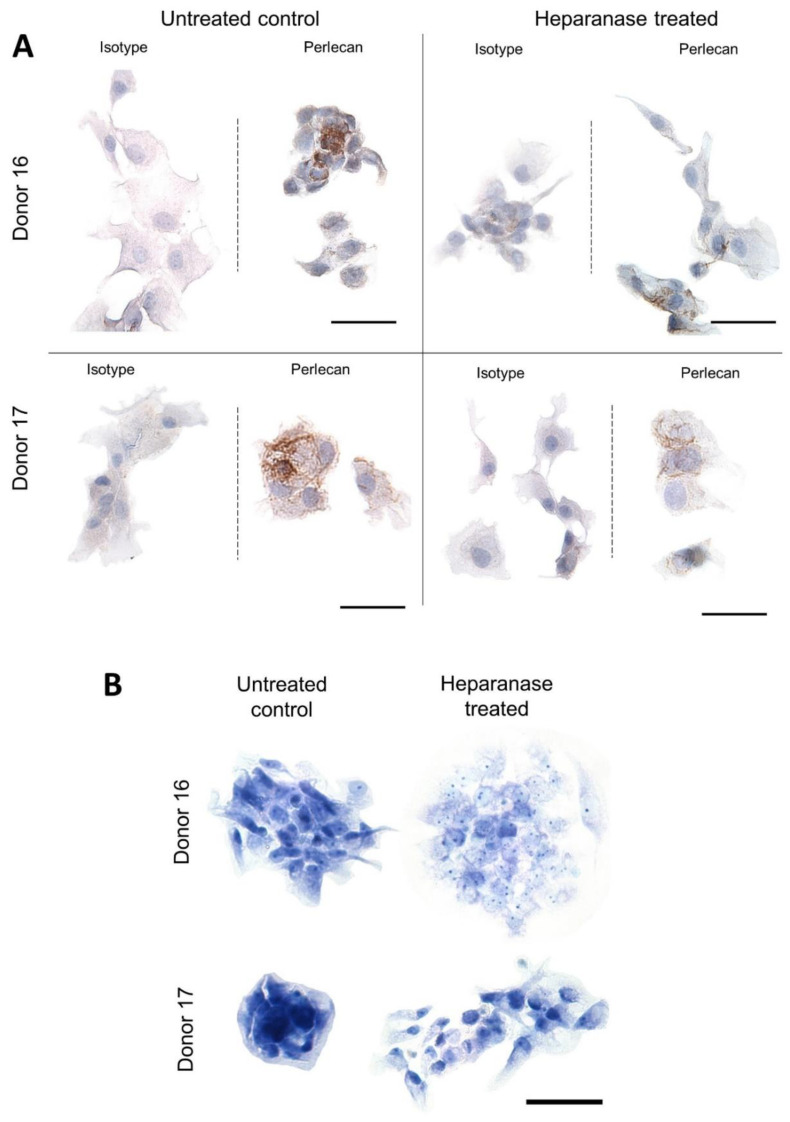
Immunocytochemistry and toluidine blue staining of heparanase-treated chondrocytes (two representative donor examples shown). (**A**) Chondrocytes cultured in chamber slides were treated with heparanase for 48 h and stained for perlecan (*n* = 4). Chondrocytes in untreated controls tended to remain in clusters that stained more intensely for perlecan compared to heparinase-treated chondrocytes. (**B**) Toluidine blue staining revealed no noticeable trend. However, cell clusters in both controls and heparanase conditions had stronger staining than individual cells. Scale bars = 50 µm.

There was immunostaining for perlecan in some cultured cells, some apparently in the cytoplasm and also associated with the cell membrane. This appeared strongest when cells were in clusters, which were more common in cultures without exposure to heparanase (Figure 8A).

Metachromasia with toluidine blue staining for glycosaminoglycans was mostly weak with no consistent difference in pattern between control and heparanase conditions (Figure 8B). However cell, clusters, where present, tended to have stronger toluidine blue staining.

## 4. Discussion

Cell-based therapies have shown some degree of success in restoring damaged cartilage [30,31], but no study to date has described the presence of the proteoglycan perlecan in either the natural repair or cell therapy repair of cartilage in humans. Perlecan contributes to processes that are essential to the functioning of chondrocytes such as cell attachment, differentiation and production of extracellular matrix components [12,13], which makes it an ideal candidate molecule to assess in the formation of new cartilage. There is a longstanding biological paradigm that once damaged, articular cartilage cannot heal itself. However, evidence is now mounting to indicate that actually, to a limited extent, articular cartilage does have an innate ability to repair [2,32], although the mechanism and pathways are poorly defined. To our knowledge, this study is the first to assess and compare the differences in perlecan immunolocalisation in matched patient cartilage samples that were repaired either naturally or with autologous cells, while assessing the effects of heparanase on the phenotype of human chondrocytes in vitro.

The variety of tissue morphologies observed in the repair tissues, i.e., fibrous, hyaline, fibrocartilage and a mixture of the two, demonstrates the unpredictable and variable nature of cartilage repair. Some of these differences could be donor dependent, but since the repair tissues have been collected from two different sources (one from the harvest site and the other post-treatment with cell therapy), the repair could have been the result of two different biological mechanisms. Furthermore, the lack of an identifiable pattern of morphology in donor-matched natural and cell therapy repaired tissues could be due to differences in the microenvironment of the location where these defects were found. The high incidence of vascularisation present in the naturally repaired biopsies is of concern, as in its native state, cartilage is avascular. One could hypothesise that there may be a temporary invasion of blood vessels as a means of instigating the repair processes and over time with tissue remodelling and maturation, this vascularisation may disappear. Synovial infiltrates are often vascularised and usually associated with poor cartilage repair [33], but a recent study has provided evidence of the contribution of synovial cells in the repair of cartilage surface injuries in mice [34]. Adhesions identified by MRI (which are likely to be vascularised) have been shown to correlate with better histological features of cartilage repair twelve months after ACI [35].

Perlecan was immunolocalised in the pericellular matrix in healthy cartilage, which was is in line with previous findings [16]. In contrast, the immunolocalisation of perlecan in the repair tissue differed depending upon the type of tissue morphology present, for example, in areas of hyaline cartilage, perlecan appeared to have a more “normal” pericellular appearance whereas in areas of fibrocartilage, it was more associated within the interterritorial extracellular matrix. The latter appears to resemble the disposition of perlecan observed in foetal patella, femoral condyle and tibial plateau tissues [36]. This, combined with the fact that perlecan is a marker of early chondrogenic activity [37], suggests that embryological mechanisms could be contributing to the repair of damaged adult cartilage, either naturally or post-cell therapy. This is further evidenced by the observation that in cell therapy repaired samples, perlecan is associated with better tissue morphology and increased proteoglycan content, more resembling normal, healthy cartilage. (One slight caveat in comparing this immunolocalisation between healthy and surgical samples, however, is that there was some disparity in times between ex-vivo collection and processing; for healthy donors, time to fixation was ~24 h + 2–4 h but for surgical samples it was much quicker (2–4 h).)

Fibrocartilage commonly forms in repair sites following cell therapy, at least in biopsies obtained ~12 months post-treatment [25]. Whilst the aim of cell therapy in the treatment of cartilage defects is the formation of hyaline cartilage, there is evidence that the initial repair tissue which forms is remodelled [38] and does indeed mature towards hyaline cartilage with time post-treatment [39]. The distribution of perlecan seen in our study is perhaps further evidence of this, with the more diffuse and widespread location seen in fibrocartilage resembling that of developing or rudimentary cartilages, some of which subsequently mature to form hyaline cartilage with its definite pericellular staining pattern. The strong vascular localisation of perlecan in the naturally repaired tissues is expected, confirming reports of its role in angiogenesis [40,41].

In healthy articular cartilage, collagen type III has a diffuse localisation in the territorial regions around the chondrocytes, i.e., beyond the pericellular capsule [28]. We found this pattern only in the repair tissues where some hyaline-like cartilage was present, suggesting more matured repair or regeneration. Collagen type III is often associated with collagen type I and is abundant in damaged tissues that are attempting to repair [28,42].

Collagen type VI is a microfibrillar collagen, accounting for approximately 1% of total collagen in adult articular cartilage [43]. Predominantly located in the pericellular matrix (PCM) in developing and mature cartilage, collagen type VI has been demonstrated to be integral for regulating chondrocyte swelling and contributing to the biomechanical integrity of the PCM; indeed, it also binds to the chondrocyte membrane via the RGD sequences [44,45,46,47]. During osteoarthritis, however, the localisation of collagen type VI changes to more interterritorial matrix expression, possibly reflecting increased degradation of the collagen fibrils [48,49]. The diffuse pattern of immunolocalisation of collagen type VI in the majority of the repair tissues tested in our study is similar to that of perlecan and indicates an immature PCM in regenerating cartilage. Despite our observations of collagen types III and VI immunlocalisation in repair tissue being similar to those found in OA, it is also possible that they are indicative of an immature, developing cartilage rather than degeneration. Both perlecan and collagen type VI have shown to be pivotal to the biomechanical function of the PCM [50]. As a result, and due to the ability of cartilage to detect and respond to mechanical loading, perlecan in particular could be an active participant in the loading-related aspects of cartilage repair and remodelling. Perlecan’s role of “mechanosensing” in tissue maintenance has been demonstrated in bone [51,52], while its ability to influence the elastic modulus of the PCM has been proven in cartilage [50].

Given the unique glycolytic capability of heparanase, this enzyme has been proposed to be a valuable therapeutic target in repair biology [53,54]. The fragments released from the HS by the action of heparanase are often more bioactive than the native molecule [55,56]. For example, when heparanase cleaves HS from perlecan in the basement membrane it releases bound FGF2, which promotes angiogenesis, wound healing and tumour formation [57,58]. In our study, we tested the effects of heparanase on chondrocytes in terms of cell morphology, proliferation, and the expression of surface and genetic markers. Although no noticeable difference in cell morphology was noted, chondrocytes treated with heparanase showed higher proliferation compared to the untreated controls. This finding corroborates a previous study showing a heparanase-induced increase in proliferation and migration of the ATDC5 chondrocyte cell line [59], and supports the theory that the removal of HS encourages an increase in cell proliferation. Further investigations are needed to determine whether this stimulation of chondrocytes by heparanase is reproduced in vivo, and what the pathophysiological implications are, notably in the modulation of tissue repair. One should also consider the source/s of the HS that has been depleted, as perlecan is not the only HS-containing proteoglycan found in cartilage.

The flow cytometry analysis conducted in our experiment produced the first data looking at the effects of heparanase treatment on the expression of a comprehensive panel of surface markers in human chondrocytes. Exposing human chondrocytes to exogenous heparanase did not influence the expression of either surface stem cells markers (CD70, CD90 and CD105), or chondrogenic markers (CD44, CD151 and CD166). Interestingly, another study in mice MSCs has shown similarly that the inhibition of endogenous heparanase has no effect on these stem cells markers [60].

Of the five chondrocyte populations tested for their response to heparanase, three of them showed a marginal increase in FGFR3 as assessed by flow cytometry, while the gene expression of FGFR3 was stable. This is of particular interest in the context of cartilage repair, since signalling through the FGFR3 pathway is essential to chondrocyte function during chondrogenesis. During the embryological development of cartilage rudiments, FGFR1c, FGFR2c, FGFR3c and perlecan are employed by mesenchymal cells to promote the production of extracellular matrix production [17,61,62]. FGF-18 has also been shown to signal through FGFR3 in the cartilaginous development of the human foetal spine [63]. Furthermore, a mouse knockout model revealed that the deletion of domain I in HS improved the symptoms of OA and preserved the expression of FGFR3 with disease progression [64]. We hypothesise that the positivity of FGFR3 on reparative cells in the de novo formation of cartilage could be an essential mediator of natural and CT repaired tissues. Additional work would, however, be needed to investigate this further.

Integrins are a family of cell adhesion receptors that are vital to the interactions between chondrocytes and the cartilage extracellular matrix, that is mediated through the binding of matrix components such as collagen types II and VI, vitronectin and fibronectin [65]. The heparanase treatment of chondrocytes in our study did not affect CD29, which is the β1 integrin subunit. CD29 couples with the α1 integrin subunit (CD49a) to form the α1β1 complex, and facilitates the binding of collagen types II and VI [66,67]. The reduced positivity of CD49a in four of the five cell populations treated with heparanase suggests a possible interaction between HS and the integrins that warrants further characterisation in cartilage repair. The heterogeneity of the expression of integrin subunits CD49b(α2), CD49c(α3) and the complex CD51/61(αV/β3) in response to heparanase, may be indicative of the versatility of chondrocytes when interacting with their pericellular environs and extracellular matrix. The marked lower levels in CD49a, CD49b and CD49c detected in chondrocytes from the oldest donor (donor 17) after heparanase treatment could reflect the age-induced decrease in integrins in cartilage that has been previously shown [68].

We tested the effects of heparanase on the expression of key chondrogenic genes and found no significant change in the expression of SOX9, collagen type II and aggrecan. This differed from a previous study that showed an increased gene expression for collagen type II and aggrecan after heparanase treatment, but this was using a more appropriate 3D culture system [59]; even in our monolayer system, heparanase had no inhibitory effect on chondrocytes. The marginal increase in expression of the hypertrophic genes for collagen type X and ALK-1 could be an indirect effect of the increased cell proliferation and is not conducive to the repair of hyaline articular cartilage. This observation should act as a reminder that the mechanisms triggered by the removal of HS would need to be controlled to avoid undesirable matrix formation [69].

The strong immunolocalisation of perlecan in chondrocyte cell clusters suggests that the pericellular matrix of these cells may still be intact, or at least being maintained, in some monolayer cultures with close cell contact. This finding confirms previous studies showing pronounced perlecan staining in clusters found in OA cartilage [70,71]. It was found that domain IV-3 of perlecan was responsible for chondrocyte clustering, by mediating a decrease in ERK1/2 signalling [72]. The presence of perlecan persisted despite after the assumed removal of HS in our study, which could indicate that the heparanase-induced response from chondrocytes is due to the loss of HS from perlecan, and not perlecan itself. Such observations were made in a mouse study where a *Hspg2* exon 3 null strain continued to produce perlecan without the native HS [13].

The present study is not without its limitations. For example, we acknowledge that a bigger sample size would make this study more robust; however, we are confident, based on our experiences, that the tissue morphologies presented here are in line with our previous observations. The naturally and cell therapy repaired tissues that we studied formed at different locations in the joint. This may have limited the direct comparison of the two cartilages, for instance due to differences in biomechanical forces betweeen different regions of the knee joint [73,74]. Regarding the in vitro cell experiments, chondrogenic differentiation may have provided additional insight into the effects of heparanase on chondrocyte function. It is also important to note that the enzymatic activity of heparanase is not specifically targeted to the HS on perlecan and that other HS proteins such as agrin, syndecan 1 and syndecan 4 may also be affected by heparanase [75,76]. This study does not identify an exact pathway or mechanism per se whereby perlecan influences cartilage repair, but it does indicate that it appears to be an integral player and so worthy of further investigation.

## 5. Conclusions

To conclude, we demonstrate that the HS proteoglycan, perlecan, is clearly present in repair tissue formed both via cell therapy repair of chondral defects and also naturally occurring repair tissue. The localisation of perlecan, as well as type III collagen, which is often found in developing or repairing tissue, is more diffuse for both molecules in the fibrocartilaginous tissue which forms initially, than in the more mature repair tissue. This more mature repair tissue has morphology resembling hyaline cartilage with has more of the typical cell-associated staining pattern seen in adult articular cartilage. The co-localisation of perlecan and collagen type VI and its biomechanical role in the PCM in repair cartilage remains unclear and further research could reveal a key mechanism that incorporates the different loading forces in the articular joint. The strong perlecan staining observed in chondrocyte clusters could be mediated via its domain IV-3 and the suppression of Erk1/2 signalling. We have also shown that heparanase treatment increases the proliferation of chondrocytes, without altering their phenotypical features, at least, as assessed in this study. Taken together, it is plausible to assume that perlecan has an important role in cartilage repair. Further work is required to fully comprehend how heparanase influences different types of repair, and whether this enzyme can be harnessed to enhance the quality of de novo cartilage repair in vivo.

## Figures and Tables

**Table 1:** Donor demographics and samples.

Donor.	Gender	Age	Surgical Intervention	Tissue Used (Experiments)	Tissue Location	
					Natural Repaired	Cell Therapy Repaired
1	F	42	Follow-up arthroscopy	Naturally and cell repaired cartilage (IHC)	Central Trochlea	LFC
2	M	22	Follow-up arthroscopy	Naturally and cell repaired cartilage (IHC)	Central Trochlea	MFC
3	M	41	Follow-up arthroscopy	Naturally and cell repaired cartilage (IHC)	Central Trochlea	LFC
4	M	29	Follow-up arthroscopy	Naturally and cell repaired cartilage (IHC)	Central Trochlea	Patella
5	M	30	Follow-up arthroscopy	Naturally and cell repaired cartilage (IHC)	Central Trochlea	Patella
6	M	34	Follow-up arthroscopy	Naturally and cell repaired cartilage (IHC)	Central Trochlea	MFC
7	F	36	Follow-up arthroscopy	Naturally and cell repaired cartilage (IHC)	Central Trochlea	Patellar
8	M	51	Follow-up arthroscopy	Naturally and cell repaired cartilage (IHC)	Central Trochlea	MFC
9	M	37	Follow-up arthroscopy	Naturally and cell repaired cartilage (IHC)	Central Trochlea	Patella
10	M	43	Follow-up arthroscopy	Naturally and cell repaired cartilage (IHC)	Central Trochlea	Trochlea
11	Unknown	21	Cadaver	Healthy cartilage (IHC)	MFC	
12	Unknown	30	Cadaver	Healthy cartilage (IHC)	MFC	
13	Unknown	40	Cadaver	Healthy cartilage (IHC)	MFC	
14	Unknown	50	Cadaver	Healthy cartilage (IHC)	MFC	
15	M	63	Cadaver	Healthy cartilage (IHC)	MFC	
16	M	71	TKR	Chondrocytes (heparanase treatment, FC, RT-qPCR)	LFC/MFC	
17	F	81	TKR	Chondrocytes (heparanase treatment, FC, RT-qPCR)	LFC/MFC	
18	F	51	TKR	Chondrocytes (heparanase treatment, FC, RT-qPCR)	LFC/MFC	
19	M	74	TKR	Chondrocytes (heparanase treatment, FC, RT-qPCR)	LFC/MFC	
20	M	22	Cadaver	Chondrocytes (heparanase treatment, FC, RT-qPCR)	LFC/MFC	

FC = flow cytometry, IHC = immunohistochemistry, TKR = total knee replacement, ACI = autologous chondrocyte implantation, LFC = lateral femoral condyle, and MFC = medial femoral condyle.

## Data Availability

Data available on request due to restrictions eg privacy or ethical.

## Supplementary Materials

The following are available online at https://www.mdpi.com/2218-273X/11/1/92/s1, Figure S1: Representative negative controls for immunohistochemistry studies.
