# Supplementary material for: Perlecan in the Natural and Cell Therapy Repair of Human Adult Articular Cartilage: Can Modifications in This Proteoglycan Be a Novel Therapeutic Approach?
Source: Biomolecules. 2021 Jan 13;11(1):92. doi: 10.3390/biom11010092 (PMC7828356; doi:10.3390/biom11010092)
Supplement: Supplementary file 1 [file biomolecules-11-00092-s001.pdf]

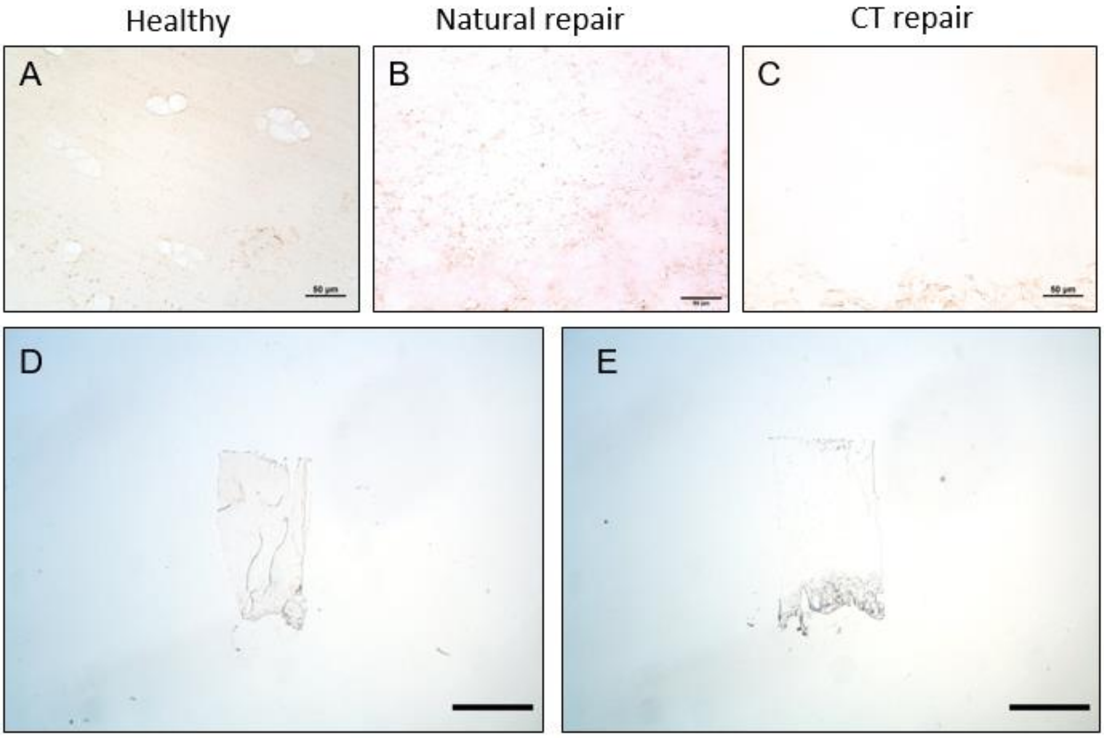

Figure S1: A-C) Representative negative controls using an isotype matched murine IgG1 (purified from hybridoma culture supernatant) for the immunohistochemistry of perlecan (as shown in Figure 2) for healthy (A), naturally repaired (B) and cell therapy repaired (C) cartilage. Images shown are for Donors 13, 2 and 9, respectively. Scale bars = 50µm. **D-E)** Representative negative controls for the immunohistochemistry of collagen types III (D, isotype matched murine IgG1) and type VI (E, normal rabbit serum) in healthy cartilage (Donor 15) as shown in Figure 4. Scale bars = 500µm.
